# Statistical analysis supports UTR (untranslated region) deletion theory in SARS-CoV-2

**DOI:** 10.1080/21505594.2022.2132059

**Published:** 2022-10-10

**Authors:** Zhaobin Xu, Dongying Yang, Liyan Wang, Jacques Demongeot

**Affiliations:** a Department of Life Science, Dezhou University, Dezhou, China; b Department of Medicine, Dezhou University, Dezhou, China; c Laboratory AGEIS EA 7407, Team Tools for e-Gnosis Medical, Faculty of Medicine, University Grenoble Alpes (UGA), La Tronche, France

**Keywords:** SARS-COV-2, deletion of the untranslated region, nucleic acid degradation system, viral toxicity

## Abstract

It was noticed that the mortality rate of SARS-CoV-2 infection experienced a significant declination in the early stage of the epidemic. We suspect that the sharp deterioration of virus toxicity is related to the deletion of the untranslated region (UTR) of the virus genome. It was found that the genome length of SARS-CoV-2 engaged a significant truncation due to UTR deletion after a mega-sequence analysis. Sequence similarity analysis further indicated that short UTR strains originated from its long UTR ancestors after an irreversible deletion. A good correlation was discovered between genome length and mortality, which demonstrated that the deletion of the virus UTR significantly affected the toxicity of the virus. This correlation was further confirmed in a significance analysis of the genetic influence on the clinical outcomes. The viral genome length of hospitalized patients was significantly more extensive than that of asymptomatic patients. In contrast, the viral genome length of asymptomatic was considerably longer than that of ordinary patients with symptoms. A genome-level mutation scanning was performed to systematically evaluate the influence of mutations at each position on virulence. The results indicated that UTR deletion was the primary driving force in alternating virus virulence in the early evolution. In the end, we proposed a mathematical model to explain why this UTR deletion was not continuous.

## Introduction

Since the outbreak of COVID-19, the virus has caused a very high number of infections and deaths worldwide and has become the most prominent public security crisis in the world since World War II [1–3]. Unlike SARS, MERS and other coronaviruses, SARS-CoV-2 did not naturally disappear but turned out to be a global pandemic and is very likely to coexist with our human beings for a long time. We hypothesize that the epidemic duration of certain RNA virus is not only contributed by the depletion of susceptible population. Instead, the elimination of RNA viruses might also be influenced by the deterioration of its own virulence. The virulence degradation might be partly contributed by the deletion of its genome, which we referred as “untranslated region (UTR) deletion theory” in this research.

The untranslated regions of viruses are untranslated segments located at both ends of their genome. Its 5”ends generally exist as internal ribosome entry sites (IRES) during translation. Although the deletion of UTR regions of viruses will not affect the properties of encoded proteins, it will significantly affect their translation efficiency. This deletion would reduce virus replication efficiency and toxicity, which has been elucidated in many viruses such as coxsackievirus [4–6] and HCV [7–9]. Although the function of the UTR region of coronavirus has been studied [10,11], and some scholars have pointed out its essential role in virus replication [12], no one has found that UTR region deletion will naturally occur during SARS-CoV-2 infection. In 2020, we proposed a hypothesis that some RNA fragments from SARS-CoV-2 genome might hybrid with messenger RNAs in human cells, such as Human haemoglobin beta-subunit (HBB), this hybridization might inhibit the normal cellular activity and might cause a deletion in viral UTR region as well [13]. In 2021, Farkas et al. further demonstrated that the viral 3” UTR engaged a significant deletion and displayed increased viral diversity [14]. This deletion may correspond to lower virus activity. In short, UTR deletion theory means that for RNA viruses, their UTR could be irreversibly deleted by the host nucleic acid degradation system, which could lead to the declination and an eventually vanish of their virulence.

Several phenomena during the early epidemic stage indicated there was uncovered intrinsic mechanism that significantly influenced the virulence evolution of SARS-CoV-2. It was noticed that the virulence of SARS-CoV-2 engaged a fast decline soon after the epidemic outbreak. Its mortality dropped from 7% in Wuhan to less than 1% in other regions in China [15]. Most of the death in Wuhan is contributed by early infections. The later infection turned out to be very mild or even asymptomatic. For instance, in the very late stage of Wuhan epidemic in July 2020, more than 300 positive cases were diagnosed but they were all asymptomatic, which revealed a speedy virulence degradation [15]. Of greater interest is that the genome sequence results implied that there were negligible differences compared to the original strain. This rapid virulence degradation can be also recaptured in the fast mortality declination globally. SARS-CoV-2 replication is very conservative, and the mutation rate is meagre, so it is difficult to evolve a mutant with sharp virulence declination in a short time [16–18]. Therefore, the virulence alternation in the early epidemic stage cannot be convincingly explained by point mutation theory. No new variants and clades emerged at that time, which was a completely different story when we compared the virulence of those latterly emerging strains such as Delta or Omicron. Another confusing phenomenon is that the SARS-CoV-2 infection displayed an extreme variability in clinical outcomes during the early epidemic [19–21]. The infection could be very deadly, severe, mild, or even asymptomatic. This reflects a high degree of heterogeneity in the virus genome in the early epidemic phase, even with no mutational effects. All those erratic scenarios above evoked us to excavate an intrinsic underlying mechanism.

We proposed the UTR deletion theory from the following six directions: first of all, it was confirmed that the genome length of SARS-CoV-2 managed a significant decline in the early stage of the epidemic. Then, employing sequence alignment, we proved that there was an evolutionary relationship between clades with different UTR lengths. Viruses with long UTR fragments can evolve into viruses with short UTR fragments. There was an irreversible legitimate relationship between them rather than a sibling relationship after parallel evolution. In the third part of the results, a strong positive correlation between mortality and UTR length was established. In the fourth part of the results, a good correlation between clinical outcomes and the viral genome length was also discovered by a systemic statistical analysis. In the fifth part of the results, a genome-level scanning was systematically performed to evaluate the influence of genetic mutations on virulence. The results further demonstrated the importance of UTR on SARS-CoV-2 virulence. In the sixth part of the results, we tried to explain why the UTR deletion in SARS-CoV-2 is discontinuous and unsustainable by constructing a mathematical model. It mathematically demonstrated why the UTR of SARS-CoV-2 did not undergo continuous deletion to a complete extinction like SARS and MERS but could exist in a relatively stable length plateau for quite a long time.

## Results

### Investigation of the genome deletion in SARS-COV-2

The sequence information of SARS-COV-2 is booming with time. As of 20 August 2022, more than 12.23 million SARS-COV-2 genomes information had been uploaded to the GISAID database. We extracted those sequences information from the GISAID database. After filtering out the samples with uncomplete genomes, the genome length distributions at different time points were shown in Figure 1a.

**Figure 1a.**
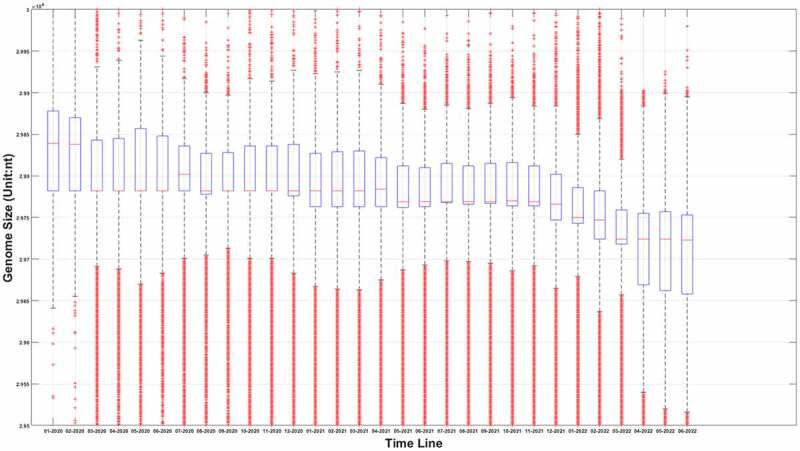
(A) the boxplot of SARS-COV-2 genome length distribution at different time points. The median is represented by the horizontal bar inside rectangles. The interquartile range box represents the middle 50% of the data. The whiskers extend from either side of the box. The whiskers represent the ranges for the bottom 25% and the top 25% of the data values, excluding outliers. (B) the average and the standard deviation of SARS-COV-2 genome length at different time points. The average value is marked as the red circles. Standard deviation of its genome length at different months is represented as the ranges marked in red. The emergence timeline of Delta and Omicron is also marked.

It can be seen from Figure 1a that virus genomes at different sample collection months have been deleted to varying degrees. Interestingly, the deletion is remarkable when we compared the original strains which mainly collected in January and February of 2020 to those latterly emerging strains. As for the average value, it can be seen from Figure 1b that this downward trend is not linear. Sometimes its length will increase slightly. The upcoming of Delta reduced its average length, and the appearance of Omicron in the later period also greatly reduced the average length of its genome. However, different from the genome attenuation in early months of 2020, the attenuation of Delta and Omicron genomes is not only due to the further deletion of the UTR, but also due to the deletion of internal coding region. For example, compared with the original strain, Delta mutant has over 10 deletions in the coding region with 6 deletions in S gene [22], and for Omicron, this number has increased to 40–60 after a sequence alignment. The deletion of the coding region is not as frequent as the loss of UTR region, but it is also a common phenomenon for later strains. We suspect that this loss may be caused by the degradation system related to endonuclease activity of the RISC (RNA-induced silencing complex) in the host. Figure 1 does not fully reflect the distribution of virus genome length at different times; it only reflects the changing trend of an average value. The genome length distribution of SARS-CoV-2 was presented in the supplementary materials from Figure S. 1.A to Figure S. 1.R. It was worth noting that the distribution of viral genome length at different time points was neither normal nor average distribution. The distribution spectrum was much like a chromatogram with chromatographic peaks around 29900nt and 29780nt. Therefore, it can be inferred the deletion probability was different at different length node.

**Figure 1b.**
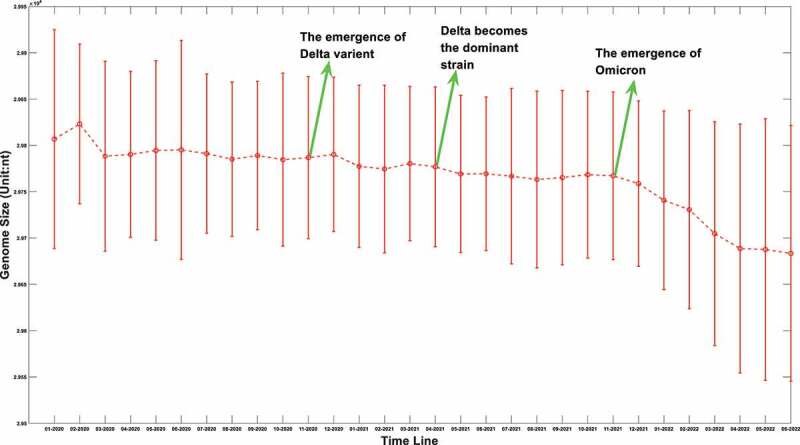
(Continued).

It was hard to follow the same pipeline in this study to SARS and MERS since the data was very limited. For instance, for MERS, there were only 618 complete sequences data in NCBI database and only a few for SARS. It was not feasible to sequence those pathogens at that time. Besides that, since it only emerged temporarily, there were not enough samples to be studied. However, it was reasonable when we claimed that SARS and MERS might engage a severe and quick UTR deletion. When we compared the dispersion of the genome length distribution between MERS and SARS-CoV-2, it can be noticed that the genome length of MERS is much more varied with larger covariance (≈475nt) than that of SARS-CoV-2 (≈73nt). The genome length differences in MERS are mainly contributed by the UTR deletion. It was indicated that the UTR of MERS engaged a much more serious deletion compared to SARS-CoV-2. Unluckily, we did not obtain sufficient data to perform a similar study on SARS.

### Bioinformatics analysis indicates UTR deletion in SARS-COV-2

In part 2.1, it was displayed that the genome length of the SARS-COV-2 population was changing with time. However, it is not plausible to affirm there were deletions in the evolution because there may be a possibility that viruses of different lengths may evolve independently. Therefore, it is necessary to provide more evidence to prove the genetic relationship among those strains with varying lengths of UTR. To verify the deletion of the UTR region in SARS-COV-2, we randomly selected the sequencing data collected before 15th-March, 2020 in the NCBI VIRUS database. The early sequencing data is chosen because the early sequences contain a higher proportion of viruses with long UTR lengths. One hundred and seventy-eight long sequences (29903nt) and 108 short sequences (29782nt) were selected to study their evolutional relationship. All those sequences have high sequence quality. Multiple comparisons were performed to determine the evolutionary relationship using a polygenetic analysis approach with each one aligned with the rest sequences. If there is a parallel evolutionary relationship between the short UTR clade and the long UTR clade, the sequence homology within the same clade will be significantly higher than between them. In contrast, if the short UTR virus is derived from the deletion of long UTR virus, the sequence similarity within long UTR clade is the highest. The sequence similarity between long UTR clade and short UTR clade would be the second-highest, while the sequence similarity of within those short UTR sequences is the lowest. It will be an opposite trend if the ancestor is a short UTR clade. The details in sequence alignment are described in section 3.2. Pair-wise sequence comparison results are shown in Table 1.

**Table 1.** Statistical characteristics of mutation score of two different length groups.

Pair-wise sequence mutation score	Among 29903nt	Between 29903nt and 29782nt	Among 29782nt
Mean Value	8.4651	10.0457	10.9576
Standard Deviation	4.8509	5.5880	6.3711
Max Value	40	54	52
Min Value	0	0	0
Sample Size	15753	19224	5778

It can be seen from Table 1 that the mutation score (8.4651) within long UTR sequences is significantly lower than that between those two clades (10.0457). Meanwhile, the mutation score between those two clades is significantly lower than that within short sequences (10.9576). In another way, the sequence similarity within long UTR sequences is significantly higher than that between long UTR and short UTR clades (Kolmogorov–Smirnov test: p = 1.07 10^−135^), while the sequence similarity between those two clades is considerably higher than that within short sequences (Kolmogorov–Smirnov test: p = 8.6 10^−22^). Therefore, we can prove that the virus with short UTR length originates from the deletion of the long sequence virus, and this process is irreversible. This legitimate relationship is difficult to explicitly observe through wet experiments.

### Statistical analysis of SARS-COV-2 mortality and UTR length suggests a strong correlation exists between UTR length and SARS-COV-2 toxicity

In the above two parts, it was statistically proved the deletion of the UTR in SARS-COV-2. In the present part, a possible relationship between the length of the UTR and virus activity is further surveyed. The new cases incidence and death rate are closely related to the vaccine coverage (see for example [23] for New-York city). Vaccination proportion was significantly increased after June 2021 globally. Therefore, the low death rate in the late time might also be contributed by the vaccine implementation. It is not appropriate to integrate the data in the late months. Although it was observed in Figure 1a that the average genome length of Delta and Omicron is continuously truncating, their UTR length remains at a relatively stable level. Many deletions happened in the coding region. For instance, there are seven amino acid deletions in the coding region of Omicron when it was compared with the original strain [24]. We observed the UTR deletion has mainly happened during the early epidemic. Therefore, only the first 18 months were selected when we studied the Pearson correlation between UTR and mortality.

**Figure 2.**
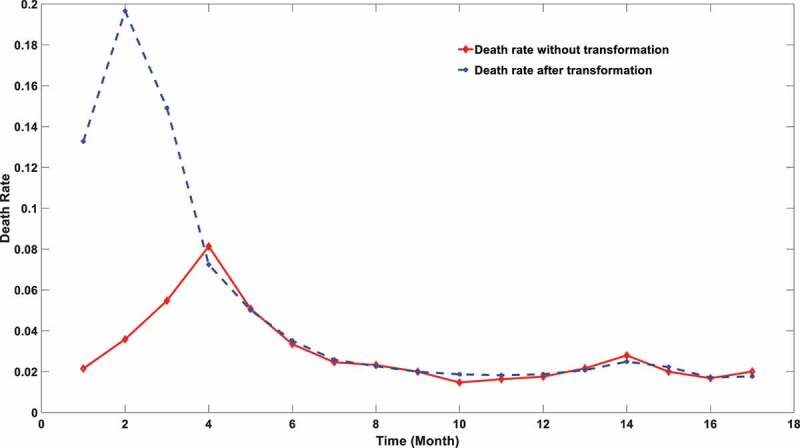
SARS-COV-2 mortality calculated using two different approaches. The red line stands for the mortality using death data explicitly. The blue stripe stands for the mortality calculated after transformation.

According to the data from a SARS-COV-2 surveillance database [25], the worldwide death toll can be extracted. The monthly worldwide mortality dynamics can be further derived by distributing the overall infection number per month. However, this simple indicator, which is widely used in the majority of epidemic surveillance websites, has a significant defect. The reason is simple but vital: death lags behind the diagnosis. People might find a low mortality rate in the early stages of the COVID-19 outbreak (late 2019 to March 2020), followed by a sharp increase. However, it did mean the early virus strain is milder because death lags much behind the diagnosis. There is a certain lag in the occurrence of death time. Viruses with strong virulence would not lead to an immediate increase in death rate in the current month but push the rise in death rate in the next one to two months after because it often takes a long medical cycle from hospitalization to death. This average cycle takes about 20 days [26]. Therefore, a more reliable indicator that can accurately reflect the virulence change is not the death rate but the future death rate of the infections at a specific time interval. For instance, if 1000 people get infected on a certain day, we want to figure out how many deaths would be among those 1000 infections instead of the number of deaths on a precise future day. However, it is tough to trace the destiny of infections. Therefore, a transformed death rate is derived after applying a gamma distribution between the diagnosis and death, which was proposed by Brazilian researchers [27]. The reported daily death number was transformed into the future death of infection during a certain time period. The details of this approach are described in Method 3.3. Figure 2 shows apparent differences in the changes in mortality with time calculated by two different statistical methods, and the changes of virus toxicity can be more effectively reflected using the transformed mortality.

It can be noticed from Figure 2 that the virulence of SARS-CoV-2 engaged a significant declination during the first few months of the pandemic. The global mortality rate after transformation reached a peak in February 2020, which is close to 20%. The virulence experienced a remarkable declination soon after this summit. It was widely reported that the mortality of SARS-CoV-2 may be subject to many confounding factors, especially vaccination coverage. However, the rapid deduction of mortality in the first few months is not associated with the application of vaccine since no vaccine was available at that period. The virulence degradation can be hardly attributed to the mutation effect because no novel variants were matured during that period. Therefore, we hypothesized the early virulence attenuation might be triggered by the deletion of the viral UTR length.

To further prove that, a Pearson correlation coefficient between the transformed mortality and the genome length is calculated. A strong correlation was exposed with a correlation factor equal to 0.7293. Using the ratio of long UTR in the overall population as a new benchmark, an even stronger correlation was discovered as shown in Table 2.

**Table 2.** Pearson correlation between genome length and death rate at different threshold sets.

Threshold Set	Pearson Correlation factor
>=29850nt	0.8191
>=29855nt	0.8082
>=29860nt	0.7980
>=29865nt	0.8371
>=29870nt	0.8508
>=29875nt	0.8217
>=29880nt	0.8351

It can be seen from Table 2 that there is a strong positive correlation between the ratio of high-length strains and the mortality rate of SARS-COV-2. When the long UTR benchmark is set to be longer than 29870nt in the entire samples, the Pearson correlation coefficient can exceed 0.85. It indicated the deletion of UTR might drive the rapid virulence attenuation during the first few months of the pandemic evolution.

### A strong correlation was found between UTR length and clinical outcomes

**Figure 3.**
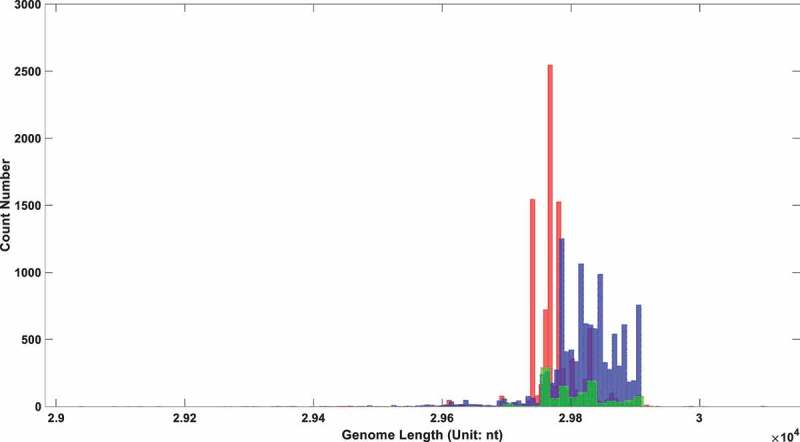
The genome length distribution of SARS-COV-2 in three different types of patients. The red box stands for symptomatic patients; the blue one stands for hospitalized patients; the green one stands for asymptomatic patients.

In the above three parts, it was demonstrated that the UTR in SARS-COV-2 experienced a distinctive deletion, and there was a notable statistical correlation between its UTR length and the virus toxicity. To further establish the relationship between UTR length and virus toxicity, sequences with different clinical symptoms were selected to perform a significant test in length. Patients were divided into three categories: asymptomatic, symptomatic, and hospitalized. The genome length distribution of SARS-COV-2 in these three types of patients is shown in Figure 3. The statistical Kolmogorov–Smirnov test *P*-value [28] within and among each group is shown in Table 3.

**Table 3.** Heterogeneity test of SARS-COV-2 genome length among different symptom patients.

P-value	Virus genome length in hospitalized patient	Virus genome length in asymptomatic patient	Virus genome length in symptomatic patient
Virus genome length in hospitalized patient	0.5311	6.2923e-41	0
Virus genome length in asymptomatic patient	6.2923e-41	0.5395	6.3969e-117
Virus genome length in symptomatic patient	0	6.3969e-117	0.8739

As shown in Figure 3, the genome length of SARS-COV-2 in hospitalized patients is significantly longer than that in the asymptomatic and symptomatic groups. This indicated long UTR length is positively correlated with viral virulence. An interesting result is that the average viral genome length in the symptomatic group is shorter than that in the asymptomatic group. All of those intergroup differences reached a significant level, as revealed in Table 3 with *P*-value approximating zero. The differences within each group maintained a relatively low level, with all three *P*-values bigger than 0.5.

So why do symptomatic people contain shorter UTR lengths compared with asymptomatic counterparts? Asymptomatic infections should be inclined to be infected with the weakest virus, that is, the virus with the shortest genome length. Lots of asymptomatic patients reported in the database were infected in their early infection stage. They cannot be strictly categorized into the asymptomatic group. They were just asymptomatic temporarily and would turn out to be symptomatic after the incubation period. At the early infection period, the host nucleic acid degradation system had not seriously eroded the viral genome. Therefore, the viral genome length is longer than that of viruses collected in the middle and late stages of infection. This interference could explain why the average size of virus genomes in asymptomatic infected people is significantly longer than that in symptomatic infected people. It did not represent that short UTR was associated with strong clinical outcomes. Instead, it implied that the UTR deletion might result from the interaction between the virus and the RNA degradation system in host cell.

### Mutation scanning at genome level indicates UTR deletion is the major driving force in SARS-COV-2 early virulence evolution

Many pioneering attempts have been made to investigate the effect of genetic mutations on viral virulence using a systematic approach [29–34]. A typical study done by Nagy et al [30]. identified 15 mutations that enhanced viral virulence, including D614 G, P323 L, etc., through the statistical comparison among viruses with different clinical outcomes. Experimental endeavours were subsequently conducted on the D614 G mutation. However, it was shown that this mutation did not cause significant changes in virulence, although the experimental evidence [35–37] demonstrated that the D614 G mutation could significantly enhance upper respiratory virus load. The statistical analysis based on the cohort study might contradict to the experiment results.

In order to increase the robustness and reliability of our approach, two improvements were made based on the classic procedure. First is the augmentation in sampling size. The transformed global mortality data of 18 months was used as a benchmark for virulence. The chi-square analysis of two extreme conditions contained 2468 dead cases and 1386 asymptomatic cases which were extracted from 90,000 patients with clinical outcome information. The adoption of big data also benefited us in reducing the noise from other influencing factors such as age, gender, medical treatment, and so on. Many other factors played roles in influencing the clinical performance of SARS-CoV-2 infection. The inclusion of large samples could help us identify the significant differences in a single factor under the interferences of other influential factors. Second, multiple indicators were applied when we evaluated the association between mutation and toxicity. Mutation on mRNA level related to the virulence change can only be identified if all three indicators are satisfied at the same time. Those three indicators were mutation density, correlation with mortality and correlation with clinical outcomes.

The frequency and density of mutations were very important in filtering out irrelevant loci. The vast majority of SARS-CoV-2 genetic codes did not undergo markable mutations and were found to be highly conserved after phylogenetic analysis. The low mutation loci also had a good Pearson correlation factor with mortality. Therefore, a bias was removed by filtering out those low mutation loci. Conservation frequency of each locus which is mutually complemented to mutation frequency, is represented in Figure 4a. As shown in Figure 4a, the conservation frequency of UTR is significantly lower than the rest region, indicating they engaged dramatic mutations. Mutations in UTR are mainly caused by deletion rather than substitution. Most of those loci are displayed in yellow colour with high conservation scores. Only dark positions were further considered in the analysis afterwards.

**Figure 4a.**
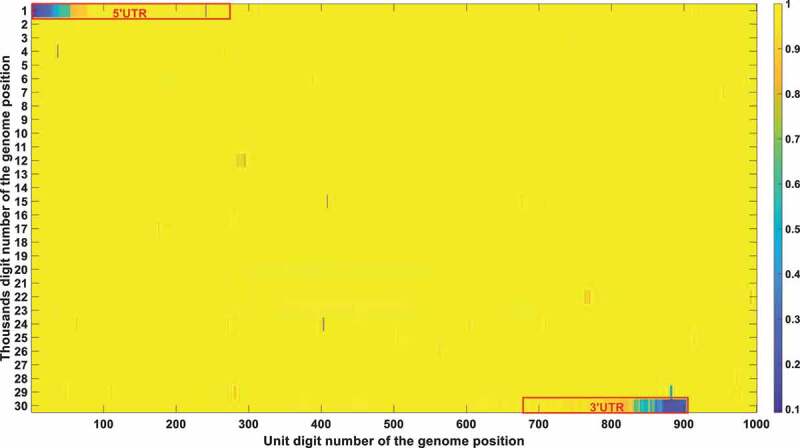
(A). Conservation frequency of each locus. The position of specific locus is marked as (the number in y coordinate-1) * 1000 + (the number in x coordinate). UTR is marked in red rectangles. (B). the Pearson correlation between the frequency of mutations in genetic variation and mortality. The position of specific loci is marked as (the number in y coordinate −1) * 1000 + (the number in x coordinate). UTR is marked in red rectangles. (C). Significance of the *P*-value of the ratio between deceased patients and asymptomatic patients calculated by chi-square test. The position of specific loci is marked as (the number in y coordinate −1) * 1000 + (the number in x coordinate). UTR is marked in red rectangles.

**Figure 4b.**
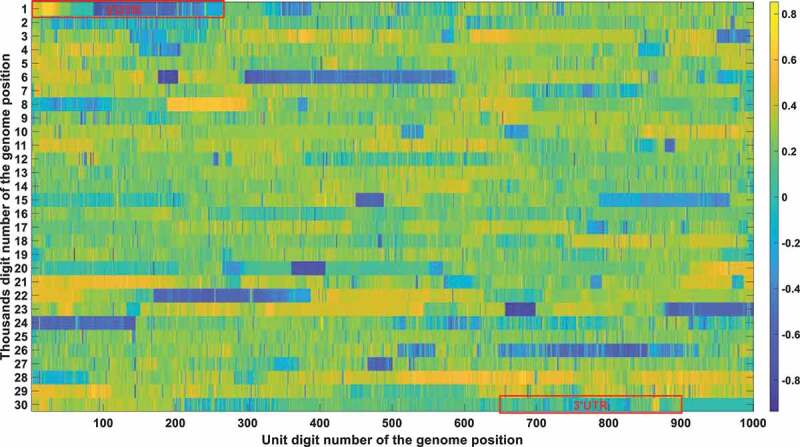
(Continued).

**Figure 4c.**
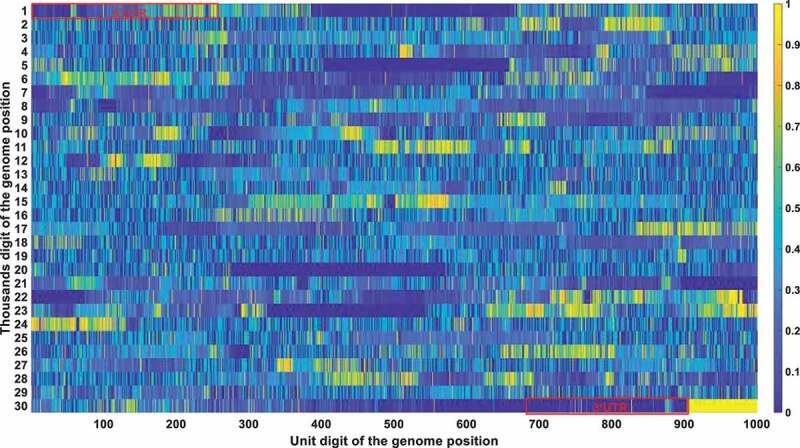
(Continued).

The Pearson correlation between the frequency of mutations and mortality was selected as the second indicator. The mutation frequency of all bases at all loci in different months can be obtained after sequence alignments. The Pearson correlation analysis was then performed to compute the correlation coefficient between mutation frequency and mortality rate. The Pearson correlation factor is positive for mutations that attenuate virulence and negative for mutations that enhance virulence, with the significance correlated to its absolute value. The Pearson correlation is displayed in Figure 4b.

The chi-square significance of the ratio between deceased and asymptomatic infections is chosen as the third indicator. Chi-square analysis is used to determine whether a single base mutation could cause a significant change in the proportion between those two. For mutations that attenuate virulence, it will cause a substantial decrease in the ratio between deceased and asymptomatic patients, with a remarkable significance value detected by the chi-square analysis. An opposite trend would be observed for mutations that enhance viral virulence. The significance of the *P*-value of the ratio between deceased and asymptomatic patients calculated by the chi-square test is represented in Figure 4c.

Integrating all those three indicators, Table 4 was derived with the loci that passed all those three filtering criteria (the mutation frequency > 20%; the Pearson correlation > 0.2; the chi-square significance *P*-value < 0.01). Its secondary structure of each loci was also marked in Table 4.

**Table 4.** Locus that meet all of the three thresholds. Specifically, the mutation frequency threshold is set to be 0.2; the Pearson correlation threshold is 0.2; the chi-square significance threshold is 0.01.

Position	Conservation score	Pearson correlation	P-value using the chi-square test with two extreme symptom groups	Inside UTR or not		Locations in secondary structure
1	0.0927	0.6907	0	Y		
2	0.1059	0.6425	0	Y		
3	0.1216	0.596	0	Y		
4	0.1278	0.6001	0	Y		
5	0.1488	0.5076	0.0004	Y		
6	0.1563	0.476	0.0029	Y		
7	0.1662	0.4416	0.0065	Y	SL1	
8	0.177	0.4307	0.0018	Y	SL1	
9	0.1817	0.3974	0.0016	Y	SL1	
11	0.1935	0.3672	0.0095	Y	SL1	
24	0.2732	0.6458	0.0044	Y	SL1	
25	0.2871	0.653	0.0001	Y	SL1	
26	0.3205	0.6346	0	Y	SL1	
27	0.3251	0.6346	0	Y	SL1	
28	0.3308	0.6381	0	Y	SL1	
29	0.3345	0.6367	0	Y	SL1	
30	0.343	0.6325	0.0008	Y	SL1	
33	0.4557	0.4655	0	Y	SL1	
34	0.4702	0.4803	0	Y	SL1	
35	0.4784	0.4845	0	Y		
36	0.4955	0.4704	0	Y		
37	0.5118	0.4479	0	Y		
38	0.5261	0.4075	0	Y		
39	0.5685	0.3208	0	Y		
40	0.5778	0.3094	0	Y		
41	0.5804	0.2977	0	Y		
42	0.5838	0.2875	0	Y		
43	0.5909	0.2652	0	Y		
44	0.5922	0.2603	0	Y		
45	0.5968	0.2476	0	Y		
46	0.5981	0.2543	0	Y		
47	0.5968	0.2499	0	Y	SL2	
48	0.6038	0.2831	0	Y	SL2	
49	0.6055	0.2763	0	Y	SL2	
50	0.616	0.2743	0	Y	SL2	
51	0.6188	0.2557	0	Y	SL2	
52	0.6215	0.2546	0	Y	SL2	
53	0.6226	0.26	0	Y	SL2	
54	0.6248	0.2498	0	Y	SL2	
11285	0.7988	0.3803	0	N		
11287	0.7994	0.3802	0	N		
11289	0.7974	0.3805	0	N		
11291	0.7967	0.3811	0	N		
11293	0.7982	0.3802	0	N		
11294	0.7964	0.3802	0	N		
11295	0.7957	0.3798	0	N		
11296	0.7938	0.3806	0	N		
29831	0.6291	0.2119	0	Y		
29833	0.6499	0.2084	0	Y		
29834	0.6521	0.2188	0	Y		
29839	0.6047	0.3783	0	Y	S2	
29843	0.592	0.3198	0	Y	S2	
29848	0.5475	0.2309	0	Y	S2	
29849	0.5271	0.2467	0	Y	S2	
29853	0.5846	0.3191	0	Y		
29854	0.4954	0.2589	0	Y		
29855	0.4692	0.2707	0	Y		
29858	0.4484	0.2083	0	Y		
29860	0.3515	0.4457	0	Y		
29861	0.2771	0.3218	0	Y	S3-B	
29862	0.3262	0.4985	0	Y	S3-B	
29863	0.3095	0.6174	0	Y	S3-B	
29864	0.46	0.6373	0	Y	S3-B	
29865	0.2757	0.459	0	Y	S3-B	
29866	0.4353	0.5005	0	Y	S3-B	
29867	0.5573	0.4274	0	Y	S3-B	
29868	0.4415	0.7173	0	Y	S3-B	
29869	0.2372	0.5257	0	Y	S3-B	
29890	0.2015	0.2203	0.0044	Y		
29891	0.2017	0.2067	0.002	Y		
29892	0.2034	0.2253	0.0032	Y		
29893	0.205	0.2399	0.0022	Y		
29894	0.2069	0.2409	0.0013	Y		
29895	0.2084	0.25	0.001	Y		
29896	0.211	0.2599	0.0003	Y		
29897	0.2121	0.2469	0.0002	Y		
29898	0.2159	0.2571	0.0001	Y		
29899	0.2211	0.256	0	Y		
29900	0.2504	0.3796	0	Y		
29901	0.2585	0.3614	0	Y		
29902	0.3304	0.2282	0	Y		

These bases were all in the UTR region except for the 11,285, 11287, 11289, 11291, 11293, 11294, 11295, and 11,296 positions. Those eight mutations are all deletions. After carefully checking those non-UTR positions, we further screened those eight positions out since those mutations started to emerge after Nov 2020. Those mutations contributed to the alpha variant firstly identified in England. They were not correlated with fatality in the first 10-month epidemic. The dominant mutation types of those UTR loci were deletions. Therefore, we can justifiably speculate that the deletion of the UTR region influenced the early evolution of SARS-CoV-2.

The structure conservation of UTR has been widely studied. It was proposed that the UTR can form multiple stem-loop secondary structures which greatly enhanced its conservation in structure. A detailed description of its secondary structure is shown in a recent review done by Shalakha Hegde et al [38]. For SARS-CoV-2, in the 5’ UTR (1–265), there are five stem-loops identified, SL1–5. SL1 was demonstrated by Vankadari et al [39]. to bind to nsp1 protein and cooperate in recruiting the human ribosome. Escors et al [40]. demonstrated that SL5, which includes the genome start codon, was a four-helix junction essential for viral packaging. It is also proposed that the existence of SL1, SL2, and SL4, but not the exact nucleotide sequences, play a more fundamental role in betacoronavirus function [41]. In the 3’ UTR, three main secondary structures were elucidated by chemical probing: bulged stem-loop (BSL), SL-1, and the highly variable region (HVR) [42]. Our study indicates the sequence conservation of its UTR is much lower than the rest regions as shown in Figure 4A. This is not contradicted to its conservation in structure. Its secondary structure formed a basis when we explained why the deletion of its UTR was not continuous in section 2.6. Meanwhile, those structure studies in UTR provided a theoretical foundation for the virulence attenuation of SARS-CoV-2. For instance, 5’UTR performs not only as IRES but is also required to evade Nsp1-mediated translational suppression [43]. The partial deletion or perturbation of its UTR is not fatal to the virus but would greatly influence its replication capacity [43,44].

Using the same approach, we did not find any locus that was significantly negatively correlated with viral virulence. That is, no mutations at any sites were found to increase viral virulence substantially. This result is contradicted to many previous statistical studies [29–34] but is consistent with the majority of experimental reports.

### Develop virus micro-amplification model considering UTR deletion effects

A mathematical model was constructed to explain the UTR deletion bottleneck in long-term evolution. As described in the introduction part, the UTR of SARS-CoV-2 might not engage a continuous declination similar to other coronaviruses such as SARS or MERS. On the contrary, its UTR length is maintained in an equilibrium state after a rapid decline. Therefore, it is very hard to be eliminated in a short time and is highly likely to assimilate into the global ecosystem.

**Figure 5.**
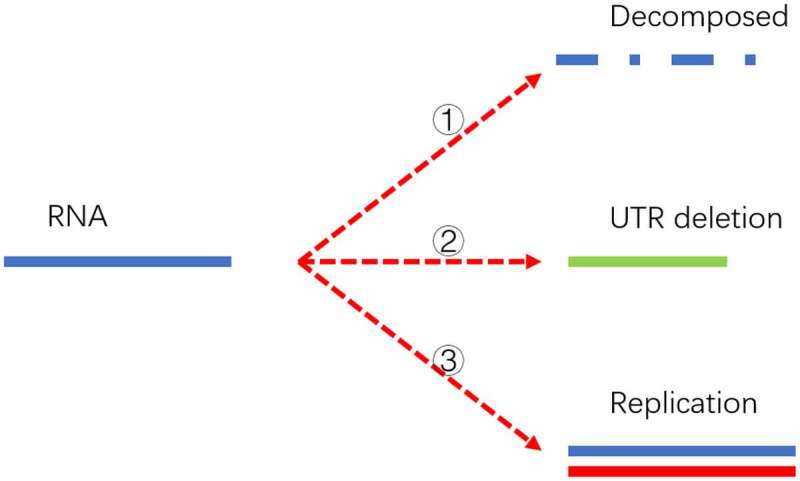
Three destinies of genome RNA in our mathematical model. The first fate is that it might be decomposed and eliminated in the host cell if it doesn’t pass the surviving threshold. The second possibility is deleting into a shorter UTR genome under the pressure of the human RNA degradation system. The shorter genome is depicted as a short solid green line. The third possibility is that it might replicate into two offspring with the template marked as a solid blue line and the new strand marked as a solid red line. The replication can be triggered if the time interval passes the replication cycle.

The mathematical model is illustrated in Figure 5.

In order to explain this process in a simple way, only two generations are explicitly drawn in Figure 5. The actual offspring’s generation would be up to several hundred to thousands. Three threshold switches are applied to simulate its proliferation process. The first controlling factor is a survival probability within a certain time interval. This survival probability is equal to the half-life of the virus which could be greatly reduced given a strong host immune response. In this mathematical model, a homogeneous host environment is applied to ensure a unique survival probability for a specific time interval. Only if the virus passes the survival threshold can it enter the next generation. The second threshold is the replication time for different strains. The virus replication time is linked to its UTR length in our model. Long UTR is more efficient in protein translation and replication, which would display a shorter replication time compared with the short UTR counterpart [12,43,44]. The relationship between replication time and its UTR length is described in section 3.5. If the virus passes the first threshold and the time interval is longer than its replication cycle, it would manifest into two viruses. If the virus passes the first threshold but the time interval is shorter than its replication cycle, it will remain in its former status. The last threshold switch is the UTR deletion probability during a specific time interval. The deletion probability is described in section 3.5. This probability can be homogeneous and independent of its UTR length. In this case as demonstrated in Figure 6a, its UTR length would engage a continuous declination. Therefore, a discriminated deletion probability is proposed by us. This hypothesis is physically supported by its RNA structure. Its RNA forms a variety of stem-loop structures, and the highly complemented area might perform as a bottleneck in the degradation by RNA exonucleases [38,45,46]. This hypothesis can also explain why the gene length distribution of the SARS-CoV-2 population does not conform to the orthographic distribution or random distribution but has significant hotspots at some specific length loci. If it passes the deletion threshold, its genome length will reduce to a lower level in the next generation. Once its genome length is truncated, it can never return to its original state. Its offspring also inherit this deletion.

**Figure 6a.**
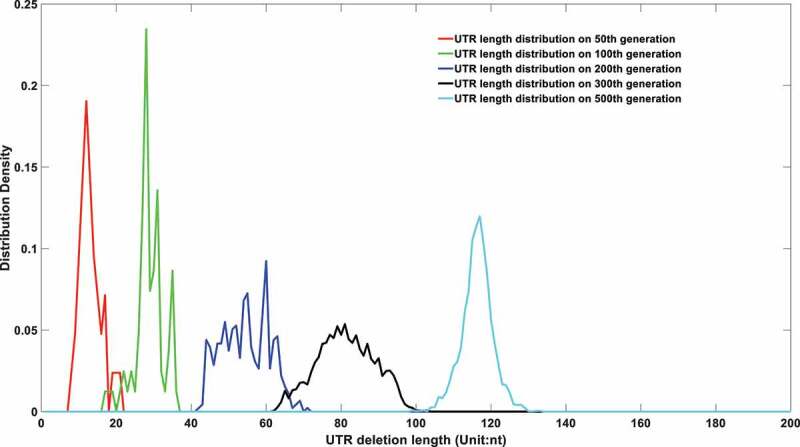
(A). UTR deletion size distribution at different generations based on undifferentiated attenuation model. 50^th^, 100^th^, 200^th^, 300^th^ and 500^th^ generations were selected to further analyse their UTR region deletion degree. 50^th^, 100^th^, 200^th^, 300^th^ and 500^th^ generations were marked in the red line, green line, blue line, black line, and cyan line, respectively. (B). UTR deletion size distribution at different generations considering reduced deletion probability at certain bottleneck points. 50^th^, 100^th^, 200^th^, 300^th^ and 500^th^ generations were selected to further analyse their UTR region deletion situation. 50^th^, 100^th^, 200^th^, 300^th^ and 500^th^ generations were marked in the red line, green line, blue line, black line, and cyan line, respectively.

**Figure 6b.**
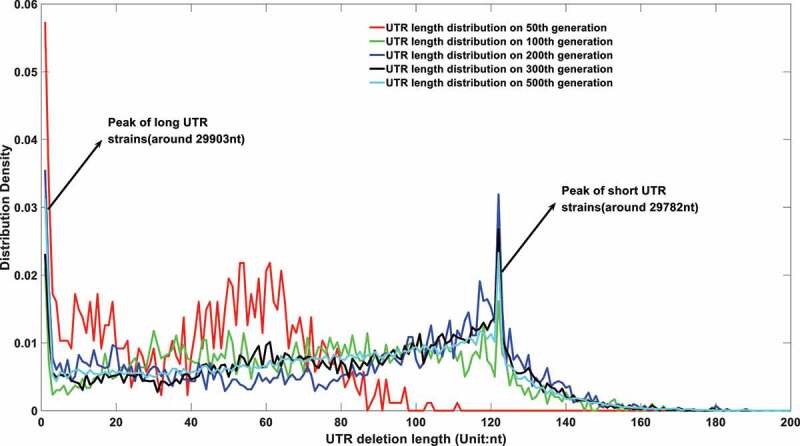
(Continued).

Those three thresholds influence the evolution of its offspring’s genome length. Although the deletion pressure is applied to all viruses at all times, it does not mean the genome length of the overall virus population would exhibit a continuous declination. An important reason is that the shorter UTR virus is deficient in self-replication, which brings a disadvantage in the quantity competition against its longer UTR counterpart.

In order to systematically prove the UTR deletion probability is correlated to its secondary structure, two models are proposed to simulate its genome length evolution under different assumptions. The first model assumes the UTR deletion is independent of its secondary structure and the deletion probability is a uniform number that is irrelevant to its current length. The results are displayed in Figure 6a.

As shown in Figure 6a, the viral genomes in different epidemic periods will be linearly deleted with time. However, the sequencing results imply that although the genome of the virus displayed a deletion trend in the first few months of the epidemic, it reached homoeostasis in the last few months of 2020. Therefore, a second model which integrates the deletion bottleneck is further proposed. In this model, the deletion probability at different loci is varied. For simplification, only three deletion probabilities are applied in this model. Specifically speaking, a small deletion probability at 29903nt genome length and an even smaller deletion probability at 29782nt genome length are adopted to simulate the evolution of its future genome length. The simulation results are displayed in Figure 6b.

It can be seen from Figure 6b that if the UTR deletion probability is largely affected by its current length, the virus population genomes will only engage a significant declination in the early epidemic period. Its UTR would reach an equilibrium state after then, which is consistent with the current sequencing results. At the same time, its genome length distribution will be concentrated at specific lengths, as shown in Figures S 1.A to 1.R. The size of the UTR is closely related to virus activity. A high mortality can be raised given a more deadly strain with long UTR length, which was the case in the early stage of the epidemic. A rapid declination in virulence of the virus might be observed when its UTR was gradually deleting in the following stage. However, UTR might form a stable secondary structure at some specific node point with a restricted deletion probability, thus forming several bottlenecks in its continuous deletion. The existence of a stem-loop structure may effectively prevent further corrosion from the host nucleic acid degradation system. Therefore, the distribution of virus genome length will not be attenuated all the time as shown in Figure 6b. The stability in genome length laid a foundation for its long-term existence.

## Methods

### Data source

All available SARS-CoV-2 (taxid: 2697049) viral nucleic acid sequences were downloaded from the GISAID virus repository (https://www.gisaid.org/) [47]. The sequences were acquired in FASTA format. Those viral sequences were selected where the entire viral nucleic acid sequence was published. As of 20 August 2022, the database we used contained 12.23 million pieces of COVID-19 data, including virus identity, sample collection location, sample collection date, genome length, genome integrity, and so on. Viruses with incomplete genomes are screened out. The specific data information and MATLAB codes are provided in the supplementary materials.

### Sequence alignment approach

In the Results section 2.3, sequence alignments were implemented to demonstrate the legitimate relationship between short UTR strains and long UTR strains. The sequence reservoir is composed of 108 short UTR sequences (29782nt) and 178 long UTR sequences (29903nt). Their sequence information is obtained from the NCBI virus database [48] by applying the date restriction as before 15 March 2020. All sequences are cut into the same length to avoid the bias caused by genome length differences. The running time is about 5s per alignment @ Intel(R) Core(TM) i9-10900KF CPU 3.70 GHz when the NWalign [49] function is utilized in MATLAB. Therefore, a faster sequence alignment approach was developed by us to compare the similarity between two sequences. Since all sequences are truncated into the same length, no insertion or deletion penalty needs to be considered in this new algorithm. This slight change greatly accelerates the computational speed by at least two orders. Specific sequence information and the MATLAB codes are provided in the supplementary materials.

### Calculation of actual death rate

As stated in section 2.3, a transformation has to be applied in order to accurately capture the virulence change through time. After the literature review, a gamma distribution between the diagnosis and the death time is further employed to calculate the transformed death toll at a certain period.

$$\Gamma \left(\alpha \right)=\overset{\infty }{\underset{0}{\int }}x^{\alpha -1}*e^{-x}dx;$$

(1) $$f\left(x\right)=\;\frac{\lambda^{\alpha }*x^{\alpha -1}*e^{-\lambda x}}{\Gamma \left(\alpha \right)};\;$$

α was set to be 2.2,andλ was set to be 0.14 [27].

Here, f(x) represents the death probability at x-th day. The real epidemic data only physically reflects the overall death per day. This death number certainly does not equal the overall death number infected on that date. Therefore, a gamma distribution together with the real epidemic mortality data helps us to trace back the overall death number infected on certain date. For instance, the real mortality data on *N*-th day is composed by a collection of infections from first day to *N*-th day, following a gamma distribution. Equation (2) calculate the contribution of the i-th day death number on the j-th day actual death toll. Equation (3) is the summation of those partial deaths, which turns out to be the overall death number among those infections that happened on j-th day.

(2) $$T_{i,j}=\frac{f\left(j-i\right)*R\left(i\right)}{\sum_{1}^{j}f\left(k\right)}\;$$

(3) $$T\left(j\right)=\;\overset{N}{\underset{i=1}{\sum }}T_{i,j}$$

R(i) is the real epidemic death number on the i-th day. $T_{i,j}$ is the contribution of R(i) on the j-th day actual overall death number. The function f stands for gamma distribution which is depicted in Equation (1). $T\left(j\right)$ is the final transformed death number caused by the j-th day infection.

The detailed MATLAB codes are provided in supplementary materials.

### Extraction of virus information based on infection symptoms

In section 2.4, a correlation was established between viral genome length and its clinical aftermath. We used the GISAID database, extracted and downloaded the patient information data by applying the filtering criteria including good sequence integrity, high sequence quality, complete patient information, and specific sampling date. Three typical outcomes, which are specifically defined as hospitalized, symptomatic and asymptomatic infections, are used in this analysis. Sequence information with 1149 asymptomatic cases, 11117 hospitalized patients, and 9626 symptomatic cases are extracted. Detailed patient information and strain information are provided in supplementary materials.

### Equations in the mathematical modelling of SARS-CoV-2 genome length evolution

In section 2.6, a micro-amplification model was developed to demonstrate there was a bottleneck in the UTR deletion process.

(4) $$L=L-fix\left(abs\left(normrnd\left(0,\theta \right)\right)\right)/n\;$$

Equation (4) is used to describe the new genome length after UTR deletion. L represents the genome length of a certain virus. $normrnd\left(0,\theta \right)$ stands for a normal distribution with mean value equal to 0 and standard deviation equal to *θ* which is set to be 10 in all simulations. Functionabs stands for the absolute value. Function **fix** stands for the smaller closest integer of the input. n is a parameter that is closely related to the deletion probability. In the first model which assumes the UTR deletion is independent of its secondary structure, n is set to be 8 for all positions. In the second model, three deletion probabilities are applied with n equal to 11 at 29903nt length, 15 at 29782nt length and 2 at other genome lengths. A larger n value represents smaller deletion probability at certain locus. The rationale behind equation (4) is if the virus can pass the surviving switch defined in equation (6), its genome length would engage a deletion with a random number following the normal distribution. We used fix function to guarantee the newly generated sequence is an integer in nucleotide number. An additional parameter n is used to provide different deletion probabilities, or deletion distributions at different loci.

(5) $$Re\_c=\frac{Re\_c_{0}}{e^{\left(L-L_{0}\right)*p}}\;$$

Equation (5) is used to calculate the replication cycle at certain genome length. In equation (5), Re_c represents the replication cycle of certain length strain. $Re\_c_{0}$ stands for the replication cycle of the full-length virus. $L_{0}$ is the genome length of full-length virus.  p is a parameter which describes the relationship between genome length and its replication efficiency which is set to be −0.006 in all simulations. Experimental evidence indicates that UTR plays an important role in initializing the transcription and replication [42,43]. Truncated UTR would lead to a lower replication efficiency in SARS-CoV-2. We use replication cycle as a measurement that reflects its replication capacity. Short replication cycle represents a strong replication capacity and vice versa. In equation (5), the newly generated viruses with UTR deletion always have a longer replication cycle compared to their ancestor strains.

(6) $$\;S=0.5^{Time_{interval}/t_{0.5}}\;$$

Equation (6) is used to calculate the surviving probability of specific virus after a fixed time interval. S represents the surviving probability of an individual virus. $Time_{interval}$ represents the time interval between two time points. $t_{0.5}$ represent the half-life of the virus. Only it passes the surviving threshold can it move into the next time point. Since the modelling followed a stochastic principle, each virus was killed randomly but with an overall surviving percentage equal to S in the population level.

Therefore, the overall process follows like below:

After a fixed time interval, if the virus passes the threshold defined in Equation (6), it survives and moves into the next life cycle. Alternatively, it would be eliminated and removed in the next time point. Once it passes the surviving switch, if the time interval is longer than its replication cycle, it would divide into two offspring with each genome length suffering a deletion following Equation (4). The newly generated offspring would have an updated replication cycle defined by Equation (5). If the virus passes the surviving switch without satisfying the replication requirement, in other words, if its replication cycle is longer than the time interval, it would not generate new offspring but remains itself. However, its genome length would also be deleted which would lead to a longer replication cycle. We mimic the population behaviours by initializing the simulation with a small number of ancestor strains with full genome length. A new virus population with different length distribution can be obtained at each time interval. A dynamic genome length evolution can be interpreted in this way as shown in Figure 6a.

## Discussion

Although the deletion of UTR has been verified in a variety types of RNA viruses [4–14], we put forward the hypothesis of UTR deletion theory in SARS-COV-2 after a systematic analysis. It was found that the length of UTR had a significant correlation to its biological activity. Viruses with short UTR had weaker replication capacity which displayed a lower mortality and a milder clinical outcome in virulence, while viruses with long UTR possessed stronger virulence. Another persuasive piece of evidence that supports the UTR deletion theory is the effect of face masks. It has been widely reported that the application of face masks would not only prevent infection but also decrease the severity of symptoms once infected [50]. However, according to the recent experimental study of the dose-effect of infection, the peak viral load has almost no relationship to its inoculum dosage. For instance, Best et al [51]. discovered that the severity of Zika infection has no relationship to inoculum dose. Our theoretical study also indicates the inoculum dosage only influences the incubation period but not the severity of the symptom [52]. The variation in symptoms upon different inoculum doses can only be explained when the inhaled viruses are genetically heterogeneous. The viral genetic heterogeneity from a specific patient can hardly derive from point mutations. It is more likely caused by the UTR deletion effect. In other words, viruses released from a certain patient are varied in UTR length with different deletion sizes. The more virus inhaled into the susceptible host, the higher chance the long UTR strain would enter into the host. This could bring a higher chance in displaying a severe clinical outcome.

Nevertheless, we should be very prudent in making the judgement that UTR deletion contributed to the early virulence attenuation of SARS-CoV-2. The mortality caused by SARS-CoV-2 infection is influenced by many complicated factors such as medical treatment and vaccination coverage [53–55]. Meanwhile, the point mutations, especially those mutations that influence the virus packing efficiency, would play critical roles in its transmission and virulence evolution in the long term, as claimed by our virulence evolution theory [56]. Therefore, a strong correlation between UTR length and its virulence is not necessarily indicating that the virulence alternation came from its UTR deletion. The deletion of UTR may only be the incidental result of evolution but not the cause of toxicity change. Explicit experimental studies need to be performed to validate this relationship further.

The evolution in its UTR length might be influenced by a comprehensive interaction between degradation and replication. In our mathematical model, it was proved the deletion probability of each locus was dependent on its secondary structure, which could lead to several deletion bottlenecks. Those bottlenecks might play an important role in maintaining its genome length. It was also demonstrated the short UTR strains were unfavourable in replication which hampered a complete UTR deletion in evolution. Therefore, for SARS-CoV-2, it is difficult to die out naturally like other coronaviruses.

The UTR deletion theory, if validated by future wet experiments, might have a positive influence on eliminating SARS-CoV-2. It was very frustrating when we realized that massive vaccination was insufficient in eliminating this epidemic. The idea of herd immunity has been challenged repeatedly by opposite reports [57–59]. On the theoretical level, our research group has also reiterated the limitations of the theory of group immunity in SARS-CoV-2 [60,61]. Because of the mutation effect of viruses and the time attenuation effect of antibodies, the idea of exterminating viruses by the vaccination paradigm is not feasible. Therefore, could we tame the virus into a milder one based on the function of its UTR? Some scholars have already attempted to develop drugs based on the inhibition of the UTR function. Some research aims to inhibit 5”UTR function by noncoding RNAs. For instance, Baldassarre et al. described the potential use of noncoding RNAs and innovative therapeutic strategies to target the 5”UTR of SARS-CoV-2 [14,62].

Natarelli et al. proposed some noncoding RNA would bind to the 5’UTR of the viral genome to inhibit its replication activity [63]. Vora et al. discovered that targeting SL1 with locked nucleic acid antisense oligonucleotides inhibits viral translation and makes SARS-CoV-2 50 UTR vulnerable to Nsp1 suppression, hindering viral replication in vitro at a nanomolar concentration, as well as providing protection against SARS-CoV-2–induced lethality in transgenic mice expressing human ACE2 [44]. It might also be applicable if we can accelerate the deletion of UTR, especially when we realize there were many bottlenecks in the natural degradation. The RNA interference, enhancement of nucleic acid exonuclease activity, or other drug treatments could also be used to expedite the deletion of the virus genome while being vigilant against too fine fragmentations of the viral genome which could cause inhibitions of the translation by hybridization of the host messenger RNAs [11]. This irreversible deletion not only can play a therapeutic role but also accelerate the evolutionary virulence attenuation at a genetic level. If the natural host degradation system cannot efficiently go over the bottleneck locus with a typical stem-loop structure, we might accelerate this process through manual intervention. It is tantalizing if we can eventually tame the virus into a harmless one.

## Data Availability

The data presented in this study are available in the supplementary materials.
